# Bimodal Effect of NKG2A Blockade on Intratumoral and Systemic CD8 T Cell Response Induced by Cancer Vaccine

**DOI:** 10.3390/cancers16112036

**Published:** 2024-05-27

**Authors:** Erika Riva, Susanna Carboni, Wilma di Berardino-Besson, Mati Moyat, Elodie Belnoue, Laetitia Devy-Dimanche, Matteo Rossi

**Affiliations:** 1 Amal Therapeutics, Fondation Pour Recherches Médicales, Avenue de la Roseraie 64, 1205 Geneva, Switzerland; 2 Boehringer Ingelheim International GmbH, 55216 Ingelheim, Germany

**Keywords:** cancer vaccination, immune checkpoint inhibitors, NKG2A blockade, CD8 T cell exhaustion, memory CD8 T cell response

## Abstract

**Simple Summary:**

Combination therapy represents an important approach for the treatment of several types of cancer. We have previously shown that heterologous prime-boost vaccination with a protein-based cancer vaccine in combination with an oncolytic virus result in improved tumor growth control and deep remodeling of the tumor microenvironment. Here, we describe the impact of the immune-checkpoint inhibitor anti-NKG2A on tumor specific immune response induced by therapeutic vaccination in mouse tumor models. We show that NKG2A blockade has a bimodal effect on cancer vaccine induced T cell response, reducing the exhaustion of tumor infiltrating antigen-specific CD8 T cells, leading to an improved antitumoral efficacy, and at the same time influencing the establishment of systemic long-term immunological memory. Our data highlight the importance of considering the diverse impact of immunotherapy on systemic and intratumoral compartments, therefore potentially influencing the clinical outcome.

**Abstract:**

Immune check-point blockade (ICB) has revitalized cancer immunotherapy, showing unprecedented efficacy despite only a narrow number of indications and with limited long-term protection. Cancer vaccines are promising combination partners for ICB to widen the patient population profiting from these treatments. Therapeutic heterologous prime-boost vaccination with KISIMA^TM^ protein vaccine and VSV-GP-TAg oncolytic virus was shown to inflame the tumor microenvironment, promoting significant infiltration of antigen-specific CD8 T cells resulting in robust antitumoral efficacy in mouse tumor models, and clinical trials are currently ongoing. Here, we report the impact of NKG2A blockade on antitumoral CD8 T cell immune response elicited by KISIMA—VSV-GP-TAg vaccination in tumor mouse models. Combination therapy significantly reduced the amount of vaccine-induced exhausted CD8 T cells infiltrating the tumor, resulting in short-term improved tumor growth control and prolonged mouse survival, while it also influenced the establishment of systemic effector memory CD8 T cell response. Taken together, these data show a compartment-dependent effect of NKG2A blockade on cancer vaccine-induced T cell immunity, increasing intratumoral T cell efficacy and attenuating the development of peripheral effector memory CD8 T cell response.

## 1. Introduction

In recent years, the combination of different immunotherapies has emerged as an encouraging approach for cancer treatment, contributing to increasing the survival of patients [1]. KISIMA^TM^-based vaccines are recombinant chimeric proteins composed of three portions: first, a cell-penetrating peptide that allows delivery to antigen presenting cells and an efficient antigen presentation; second, a multi-antigenic domain containing major histocompatibility complex (MHC) class I and II restricted epitopes; and third, a TLR2/4 agonist which provides self-adjuvant activity to the vaccine [2]. We have previously shown that KISIMA-derived protein vaccines are able to induce antigen specific T cell responses and long-lasting immunological memory, leading to a potent antitumoral efficacy in different mouse tumor models [3]. Vesicular stomatitis virus (VSV)-GP expressing lymphocytic choriomeningitis virus (LCMV) derived glycoprotein was shown to maintain its infectivity while preventing unwanted neurotoxicity [4]. VSV-GP encoding ovalbumin was shown to act as a vaccine vector inducing CD8 T cell and antibody response in mice [5]. In addition, VSV-GP was shown to highly replicate in tumor cells both in vitro and in vivo, resulting in cell lysis [6].

KISIMA—VSV-GP-TAg heterologous prime-boost is a vaccination strategy combining the protein-based cancer vaccine with the oncolytic virus expressing the same tumor antigens. We have previously shown that therapeutic vaccination with KISIMA—VSV-GP-TAg resulted in significantly improved antitumoral efficacy in different mouse tumor models [7,8]. Mechanistically, KISIMA—VSV-GP-TAg vaccination not only elicited a strong peripheral antigen-specific CD8 T cell response but also induced a deep remodeling of the tumor microenvironment (TME) by promoting a significant influx of cytotoxic CD8 T cells and cross-presenting dendritic cells and by decreasing the presence of immune suppressive cells such as M2 tumor associated macrophages (TAM-2) and myeloid derived suppressor cells (MDSCs) [7,8]. Although heterologous prime-boost vaccination was shown to significantly improve tumor growth control, a substantial proportion of intratumoral CD8 T cells became exhausted, thereby limiting efficacy [7]. In the last years, immune check-point inhibitors (ICI) have been extensively used as monotherapy or in combination for the treatment of cancer patients [9]. However, clinical studies show that only a minority of patients display an efficient and long-lasting antitumoral response, highlighting the need for novel combination strategies and molecular targets. The combination of ICI and vaccine has shown promising results, as highlighted by several preclinical studies reporting a synergistic effect in cancer models [7], supported by recent clinical data from melanoma and pancreatic cancer patients [10,11]. We have previously shown that the combination of heterologous prime-boost vaccination with anti-PD1 treatment resulted in delayed tumor growth and increased mouse survival [7].

Natural Killer group protein 2A (NKG2A) is an inhibitory cell surface receptor harboring intracytoplasmic tyrosine based inhibitory motifs (ITIMs) expressed in association with CD94 on both T and NK cells [12,13]. The receptor recognizes the non-classical class I major histocompatibility complex molecules HLA-E in humans and Qa-1^b^ in mice. HLA-E overexpression has been reported in many solid tumors and was associated with poor prognosis [13]. The binding of CD94-NKG2A to peptide-presenting HLA-E induces the phosphorylation of ITIMs in NKG2A, which eventually suppresses the activation signals generated by NKG2D and T cell receptor (TCR) signaling, resulting in decreased cytotoxicity of NK and CD8 T cells [14,15]. The disruption of the interaction NKG2A—HLA-E using a monoclonal antibody has been shown to restore NKG2A^+^ CD8 T cell functionality in vitro [16,17]. Accordingly, recent preclinical studies have highlighted the potential of NKG2A blockade as a novel approach to improve the antitumoral efficacy of cancer vaccines [18,19].

In this study, we address the effect of NKG2A blockade on antitumoral immune response elicited by KISIMA—VSV-GP-TAg heterologous prime-boost vaccination in TC-1 lung epithelial tumor model and MC-38 colorectal cancer model. We show that the combination treatment resulted in enhanced tumor regression associated with prolonged mouse survival and increased number of complete responders. Mechanistically, NKG2A blockade significantly reduced the amount of exhausted, vaccination induced, antigen-specific CD8 T cells in the tumor microenvironment whereas redirected the development of peripheral immunological memory towards CD8 T cells central memory rather than effector.

Overall, these data reveal the diverse effect of NKG2A blockade on intratumoral and peripheral CD8 T cell immune responses induced by KISIMA-VSV-GP-TAg therapeutic vaccination.

## 2. Materials and Methods

### 2.1. Mice

Six- to eight-week-old C57BL/6J female mice were purchased from Charles River (L’Arbresles, France) and were housed in individually ventilated cages in a pathogen-free mouse facility.

### 2.2. Ethic Approval

These studies have been reviewed and approved by the institutional and cantonal veterinary authorities in accordance with Swiss federal law on animal protection, under the license number GE193 issued by the Geneva cantonal authorities.

### 2.3. Tumor Cell Line

The TC-1 cell line was provided by T.-C. Wu (The Johns Hopkins Medical Institutions, Baltimore, MD, USA), while the MC-38 cell line was a kind gift from Gottfried Baier (Medical University of Innsbruck, Innsbruck, Austria). Both cell lines were cultured as previously described [7].

### 2.4. Generation of Vaccine Constructs

KISIMA recombinant protein vaccines were produced as described previously [3]. KISIMA-Mad25 contains HPV-E7 CD8 epitope whereas KISIMA-Mad46 contains three different MC-38-specific mutated neoantigens (Adpgk, Reps-1, Rpl18). VSV-GP-HPV and VSV-GP-Mad46 production have been described previously [7] and both contain the same antigenic cargo as KISIMA-Mad25 and KISIMA-Mad46, respectively. The different constructions are illustrated in Supplementary Table S1.

### 2.5. NKG2A Blocking Antibody

Anti-mouse NKG2A (InVivoMab BioXCell Cat BE0339) was diluted to 2 mg/mL in 1X phosphate buffer saline (PBS, Gibco) prior to injection. A volume of 100 µL/mouse (corresponding to 200 µg/mouse) was injected intravenously (i.v.) starting from D17 post tumor implantation, two times per week for two weeks. For the tumor microenvironment analysis study, the antibody was injected at D14 and D17 post tumor implantation.

### 2.6. In Vivo Tumor Experiments

C57BL/6J female mice were implanted s.c. with 1 × 10^5^ TC-1 tumor cells in the back. On day 6 post tumor implantation, mice were primed subcutaneously with 10 nmoles of KISIMA-Mad25 vaccine at the tail base followed by an intravenous immunization boost with VSV-GP-HPV one week later and a second boost with KISIMA-Mad25 on day 27.

Mice received 200 µg of NKG2A blocking antibody intravenously two times per week for two weeks starting from day 4 post VSV-GP-HPV injection.

For MC-38 experimental study, C57BL/6J female mice were implanted s.c. with 2 × 10^5^ MC-38 tumor cells in the back. On day 6 post tumor implantation, mice were primed subcutaneously with 10 nmoles of KISIMA-Mad46 vaccine at the tail base followed by an intravenous immunization boost with VSV-GP-Mad46 one week later and a second boost with KISIMA-Mad46 on day 27. Mice received 200 µg of NKG2A blocking antibody intravenously two times per week for two weeks starting from day 4 post VSV-GP-HPV injection. Tumor size was measured with a caliper, and mice were euthanized when tumor reached a volume of 1.000 mm^3^. Tumor volume was calculated with the following formula: V = length × length × width × Pi/6.

### 2.7. Ex Vivo Cell Preparation

#### 2.7.1. Tumors

Tumors were harvested at day 20 post tumor implantation and tumor-infiltrating leukocytes (TILs) were purified as previously described [7]. CD45^+^ cells were purified using CD45 TIL microbeads (Miltenyi, Bergisch Gladbach, Germany, Cat. number 130-110-618) following manufacturer’s protocol before being used for ex-vivo analysis.

#### 2.7.2. Spleen

Spleens were harvested, smashed through a 70 µm cell strainer, and washed with washing media (DMEM supplemented with 10% FCSand Pen/Strep). Mononuclear cells were enriched by centrifugation on a density gradient medium (Lymphoprep^TM^, StemCell Technologies, Vancouver, BC, Canada) before being used for ex-vivo analysis.

#### 2.7.3. Peripheral Blood

Blood was collected from the tail vein in heparinized tubes before being enriched via density gradient centrifugation with Lymphoprep^TM^. Cells were then used for flow cytometry staining.

#### 2.7.4. Bone Marrow

Leucocytes were isolated by flushing out bone marrow cells from femur and tibia followed by an incubation in ACK lysis solution to eliminate red blood cells. Cells were then used for flow cytometry staining.

### 2.8. Ex Vivo T Cell Stimulation

TILs or splenocytes were numerated and 1 × 10^5^ or 2 × 10^6^ cells were plated per condition, respectively. Splenocytes were incubated with E7-CD8 epitope whereas TILs were incubated with bone-marrow derived dendritic cells previously loaded with E7-CD8 peptide in presence of Golgi stop (BD biosciences, Franklin Lakes, NJ, USA) and anti-CD107a for 6 h at 37 °C, 8% CO_2_. After washing, cells were stained for cell surface antigens, fixed and permeabilized using BD Cytofix/Cytoperm^TM^ PermWash Fixation/Permeabilization kit (BD Biosciences, Cat. Number 554714). Cells were then stained for intracellular cytokines.

### 2.9. Bone Marrow-Dendritic Cells (BM-DCs)

Bone marrow-dendritic cells were isolated from the tibia and femur, cultured, and activated with LPS 100 ng/mL (Enzo Cat. number ALX-581-009-L002) in the presence of 10 ng/mL of rmGM-CSF (recombinant mouse granulocyte-macrophage colony stimulating factor) from RDSystem (Cat. Number 415-ML/CF). BM-DCs were loaded with E7-CD8 peptide for 1 h and used as antigen presenting cells for tumor infiltrating CD8 T cells functionality assay.

### 2.10. Flow Cytometry

The following antibodies were used: CD45-APC-Cy7 (clone 30-F11), CD11b-PerCPCy5.5 (M1/70), KLRG1-BV421 (2F1), NKG2A-BV650 (20d5), CD11b BV711 (M1/70), CD4 BV711 (RM4-4), CD19 BV711 (1D3), CD127-APC (SB/199), CD62L-PercPCy5.5 (MEL14), CD44 BV605 (IM7), CD69 BV786 (H1.2F3), TOX-AF647 (NAN448B) from BD Biosciences. PD-1-PE-Cy7 (29F.1A12), Tim-3-APC (RMT3-23) were from Biolegend and CD8-FITC (KT15) was from MBL. Dead cells were excluded from the analysis using LIVE/DEAD Yellow or Aqua fluorescent reactive dye (Life Technologies, Carlsbad, CA, USA). MHC-peptide multimers were from Immudex (Copenhagen, Denmark) or from MBL. The absolute number of CD8 T cells in periphery and in the tumor was quantified using CountBright^TM^ absolute counting beads (Thermo Fisher Scientific, Waltham, MA, USA). Cells were acquired using an Attune NxT flow cytometer (ThermoFisher Scientific) and results were analyzed using Kaluza (Beckman Coulter, Brea, CA, USA) software version 2.2.1. Gating strategies used for the analysis are illustrated in Supplementary Figures S6–S8.

### 2.11. Statistical Analysis

Statistical analyses were performed using Prism software version 10.1.2 (GraphPad). Mann–Whitney Student’s *t*-test or ANOVA were used depending on the experiment and group were considered statistically significant if *p* < 0.05.

## 3. Results

### 3.1. Combination Treatment with Anti-NKG2A Improves the Antitumoral Efficacy of KISIMA—VSV-GP-TAg Heterologous Prime-Boost Vaccination

**Figure 1:**
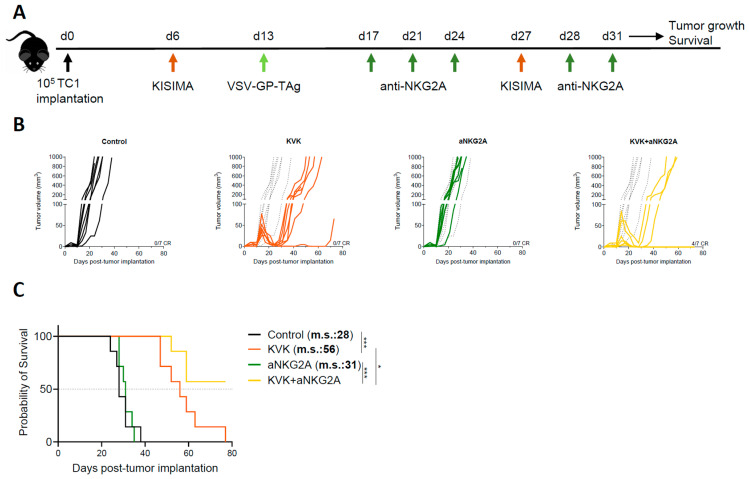
Combination treatment with aNKG2A increases the antitumoral efficacy of KISIMA—VSV-GP-TAg vaccination. (**A**) Experimental schedule. TC-1 tumor-bearing mice were vaccinated with KISIMA-Mad25 vaccine at D6 post tumor implantation followed by a boost with VSV-GP-HPV at D13 and a second boost with KISIMA-Mad25 at D27. Mice were treated with aNKG2A starting 4 days post VSV-GP-HPV boost for a total of 5 injections. Tumor growth (**B**) and survival (**C**) were monitored. CR, complete response. m.s., median survival. One representative of two independent experiments (*n* = 7). Long-rank Mantel-Cox test. * *p* < 0.05, *** *p* < 0.001.

To evaluate the impact of NKG2A blockade on the antitumoral efficacy induced by KISIMA—VSV-GP-TAg (KVK) vaccination, C57BL/6J mice were subcutaneously implanted with TC-1 tumor cells. When tumors were palpable (D6 post tumor implantation), mice were vaccinated with KISIMA containing HPV-E7-derived CD8 epitope (Supplementary Table S1). One week later, mice received VSV-GP-HPV (containing HPV-derived CD8 epitope) intravenously followed by a second boost with KISIMA at day 27 (Figure 1A). Therapeutic KVK vaccination induced significant regression of established TC-1 tumors in all treated animals (Figure 1B).

Despite the initial regression, TC-1 tumors later escaped immune surveillance and relapsed. Nevertheless, KVK vaccination significantly increased median survival by 28 days compared to untreated mice (Figure 1C). Analysis of peripheral and intratumoral E7 and VSV nucleocapsid protein (VSV-NP)-specific CD8 T cells showed significantly increased NKG2A expression in both compartments soon after vaccination (Supplementary Figure S1A,B). Interestingly, the kinetic and extent of NKG2A expression was found to differ depending on the compartment; faster up-regulation was observed in the tumor, where already 24 h post VSV-GP-HPV boost 90% of E7-specific CD8 T cells expressed higher levels of NKG2A and maintained it until at least day 14 post boost. NKG2A expression was also early upregulated by VSV-NP-specific CD8 T cells but to a lower extent, and this was not maintained over time. In contrast, on circulating E7 or VSV-NP-specific CD8 T cells NKG2A, up-regulation was observed starting only one week post boost and the increased expression was maintained one week later. Importantly, vaccination did not modulate NKG2A expression on NK cells, neither within the tumor nor in periphery (Supplementary Figure S1C,D). For combination experiments, based on the observed kinetic of NKG2A expression on CD8 T cells, mice were treated with anti-NKG2A monoclonal antibody twice weekly for a duration of two weeks starting from day 4 post VSV-GP-TAg boost (Figure 1A). While NKG2A blockade monotherapy had no impact on tumor growth nor mouse survival, in combination with KVK vaccination, it significantly increased the antitumoral efficacy resulting in 57% of complete responders and the median survival was not reached (Figure 1B,C).

Taken together, these data suggest that combination with NKG2A blockade therapy enhances the antitumoral efficacy of KISIMA—VSV-GP-TAg heterologous prime-boost vaccination.

### 3.2. NKG2A Blockade Reduces Exhaustion of Tumor Infiltrating Antigen-Specific CD8 T Cells

**Figure 2:**
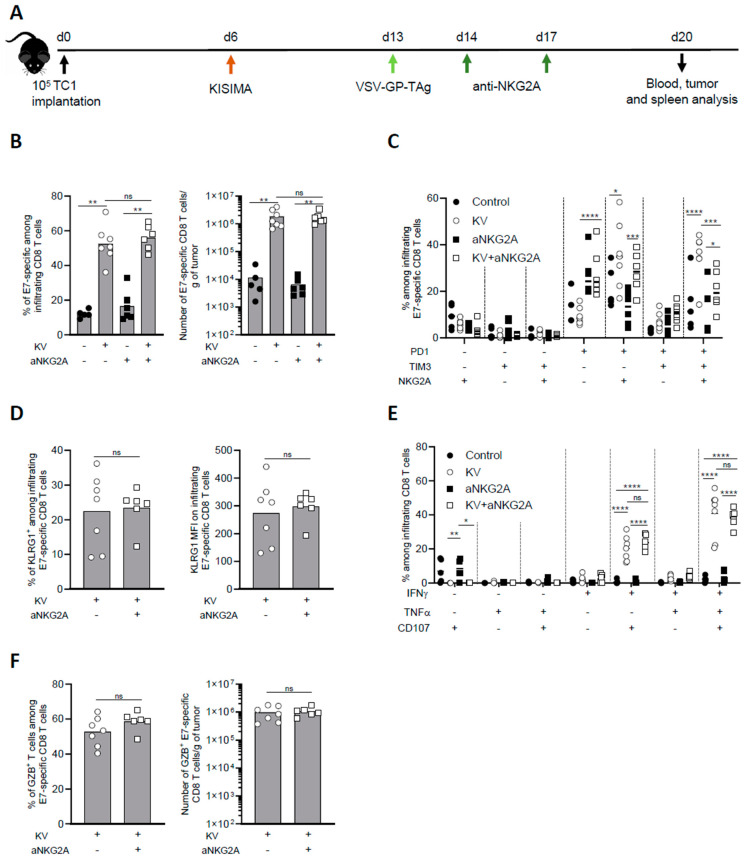
Combination treatment with aNKG2A reduces KV-induced exhaustion of intratumoral antigen-specific CD8 T cells. (**A**) Experimental schedule. C57BL/6J mice were injected subcutaneously with 10^5^ TC-1 cells at D0, vaccinated with KISIMA vaccine at D6 and with VSV-GP-TAg at D13. Mice received the first injection of aNKG2A at D14 followed by a second injection 3 days later. One week post VSV-GP-TAg boost, blood, tumor, and spleen were collected, and CD8 T cell response was analyzed by flow cytometry. (**B**) Frequency and number of intra tumoral E7-specific CD8 T cells. (**C**) Frequency of E7-specific CD8 T cell expressing exhaustion markers. (**D**) Frequency and MFI of KLRG1^+^ among E7-specific CD8 T cells in the tumor. MFI: mean fluorescence intensity. (**E**) Cytokine production by E7-specific CD8 T cells in the tumor was measured by intracellular staining after ex-vivo restimulation with E7 peptide. (**F**) Frequency and number of granzyme-B producing E7-specific CD8 T cells assessed by intracellular staining. Mann-Whitney test was used except in figure C and E where two-way ANOVA with Sidak’s multiple comparison was used (* *p* < 0.05, ** *p* < 0.01, *** *p* < 0.001, **** *p* < 0.0001). One representative of two independent experiments (*n* = 5–7).

To further investigate the mechanism of NKG2A blockade combination with KISIMA—VSV-GP-TAg vaccine, intratumoral CD8 T cell immune response was analyzed one week post boost (Figure 2A). KV vaccination resulted in high intratumoral infiltration of E7-specific CD8 T cells, which represented around 60% of all CD8 T cells, with an increase of over 100-fold compared to untreated mice. Anti-NKG2A treatment had no significant impact on the infiltration of total nor antigen specific CD8 T cells, both alone or in combination with KV vaccination (Figure 2B). Next, we addressed the exhaustion status of intratumoral CD8 T cells upon combination treatment with anti-NKG2A. As we previously reported [7], the majority of E7-specific CD8 T cells isolated from the tumor of KV-vaccinated mice expressed PD-1, TIM-3, and NKG2A, a phenotype that was reversed by NKG2A blockade (Figure 2C). Despite the decreased expression of exhaustion markers, NKG2A blockade did not increase the activation of intratumoral E7-specific CD8 T cells as shown by similar frequency and intensity of KLRG1 expression (Figure 2D). We then assessed the functionality of intratumoral CD8 T cells after ex vivo restimulation with vaccine antigen specific peptide. While in untreated mice the frequency of cytokine-producing and, in particular, of multifunctional CD8 T cells were very low, KV vaccination significantly increased the proportion of IFN-γ^+^TNF-α^+^CD107α^+^ triple positive CD8 T cells within the tumor. Surprisingly, combination with anti-NKG2A did not enhance the functionality of intratumoral E7-specific CD8 T cells (Figure 2E). In line with these results, anti-NKG2A did not significantly impact the frequency and the number of Granzyme B^+^ infiltrating E7-specific CD8 T cells induced by KV vaccination (Figure 2F).

To address whether the effect observed in the tumor microenvironment at the earlier time point (Figure 2) was maintained later in time, we analyzed the tumor microenvironment of KVK vaccinated mice alone or in combination with aNKG2A 45 days post tumor implantation. While the combination treatment did not modulate the magnitude of infiltrating CD8 T cells (Supplementary Figure S2A), it significantly reduced the frequency of PD1^+^Tim-3^+^NKG2A^+^ triple positive CD8 T cells (Supplementary Figure S2B), maintaining the effect observed at the earlier time point (Figure 2C). However, the functionality of antigen specific CD8 T cells remained unchanged upon combinatory treatment (Supplementary Figure S2C).

Altogether, these results suggest that NKG2A blockade does not enhance the infiltration and functionality of antigen specific CD8 T cells induced by KISIMA—VSV-GP-TAg vaccination, but significantly reduces their exhaustion.

### 3.3. NKG2A Blockade Dampens Peripheral CD8 T Cell Response Induced by KISIMA—VSV-GP-TAg Vaccination

**Figure 3:**
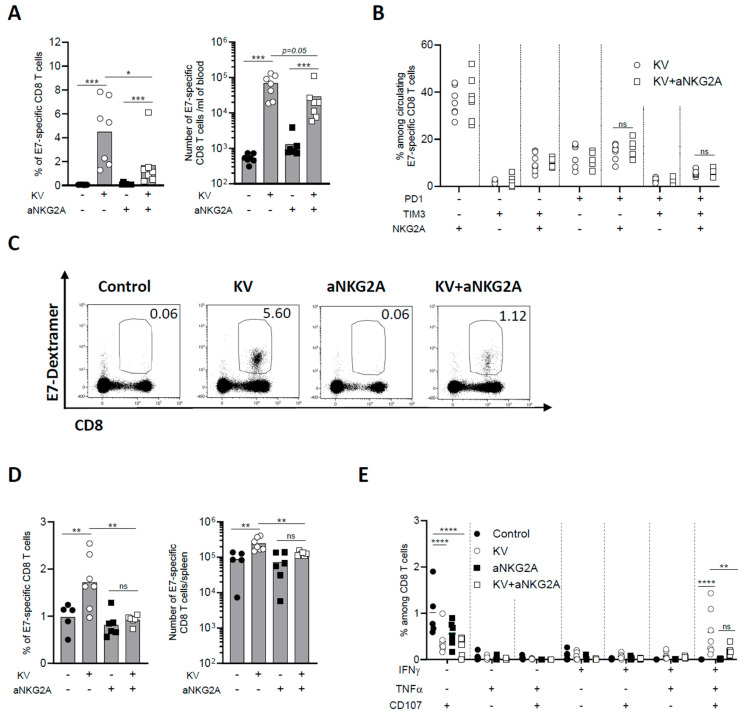
NKG2A blockade reduces peripheral CD8 T cell immune response induced by KISIMA—VSV-GP-TAg vaccination. C57BL/6J mice were injected subcutaneously with 10^5^ TC-1 cells at D0, vaccinated with KISIMA vaccine at D6 and with VSV-GP-TAg at D13. Mice received the first injection of aNKG2A at D14 followed by a second injection 3 days later. One week post VSV-GP-TAg boost, blood was collected, and CD8 T cell response was analyzed by flow cytometry. (**A**) Frequency and number of E7-specific CD8 T cells measured in the blood one week post VSV-GP-TAg boost. (**B**) Frequency of E7-specific CD8 T cells expressing activation and exhaustion markers analyzed in the blood. One representative of two independent experiments. (*n* = 7). (**C**) Representative FACS plot of E7-specific CD8 T cells in the blood. (**D**) Frequency and number of E7-specific CD8 T cells in the spleen. (**E**) Cytokine production by antigen specific CD8 T cells in the spleen was measured by intracellular staining after ex vivo restimulation with E7 peptide. Mann-Whitney test was used except for figure E where Two-way ANOVA test with Sidak’s multiple comparisons was used * *p* < 0.05, ** *p* < 0.01, *** *p* < 0.001, **** *p* < 0.0001. One representative of two independent experiments (*n* = 5–7).

The effect of KV—anti-NKG2A combinatory treatment was then assessed on peripheral CD8 T cell immune response. One week post VSV-GP-TAg boost, the frequency and number of circulating E7-specific CD8 T cells were negligeable in non-vaccinated mice, as expected from a lowly immunogenic tumor as TC-1, whereas KV vaccination induced a significant increase of antigen-specific CD8 T cells (Figure 3A). NKG2A blockade alone did not enhance peripheral immunogenicity of TC-1 tumors; however, when combined with KV vaccination, it surprisingly and significantly decreased the frequency of circulating antigen specific CD8 T cells (Figure 3A).

Differently from the tumor, the phenotype of circulating E7-specific CD8 T cells was not modulated by the combinatory treatment (Figure 3B,C). Similarly, anti-NKG2A treatment significantly reduced the frequency and number of KV-induced E7-specific CD8 T cells in the spleen (Figure 3D). Moreover, the functionality of splenic E7-specific CD8 T cells was assessed by measuring cytokine production after ex vivo restimulation with vaccine specific peptide. KV vaccination resulted in a significant portion of IFN-γ^+^TNF-α^+^CD107α^+^ triple positive antigen-specific CD8 T cells, an effect that was significantly reduced by combination with anti-NKG2A (Figure 3E). The reduction of circulating antigen specific CD8 T cells upon KV—anti-NKG2A treatment was also observed in MC-38 tumor bearing mice. Although NKG2A blockade had no impact on MC-38 tumor growth nor mouse survival of KVK vaccinated mice (Supplementary Figure S3), it significantly decreased the total number of vaccine-induced neoantigens-specific CD8 T cells, suggesting that the impact of anti-NKG2A is not antigen or tumor model dependent (Supplementary Figure S4A,B). The reduction was observed for CD8 T cells specific for each of the three neoantigens contained in the vaccine cargo and was more pronounced when the total antigen-specific CD8 T cell population was analyzed (Supplementary Figure S4C,D). Thus, these results demonstrate that while NKG2A blockade decreases the exhaustion of intratumoral CD8 T cells, it results in a significant reduction of KV-induced peripheral antigen-specific immune response.

### 3.4. NKG2A Blockade Affects the Establishment of Long-Term Antigen-Specific CD8 T Cell Memory Response Induced by KISIMA—VSV-GP-TAg Vaccination

**Figure 4:**
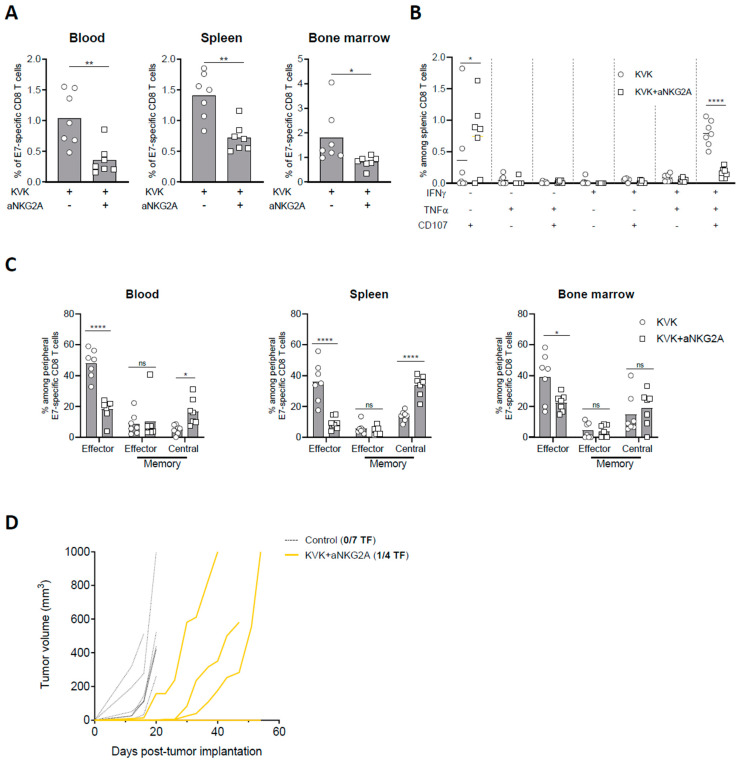
NKG2A blockade dampens long-term protection elicited by KISIMA—VSV-GP-TAg vaccination. TC-1 tumor-bearing mice were vaccinated with KISIMA-Mad25 vaccine at D6 post tumor implantation followed by a boost with VSV-GP-HPV at D13 and a second boost with KISIMA at D27. Mice were treated with anti-NKG2A starting 4 days post VSV-GP-HPV injection for a total of five injections. Mice were sacrificed at D45, tumor, spleen and bone marrow were harvested, processed and CD8 T cell immune response was analyzed by flow cytometry. (**A**) Frequency of E7-specific among CD8 T cells in blood, spleen, and bone marrow. (**B**) Cytokine production by antigen specific CD8 T cells in the spleen was measured by intracellular staining after ex-vivo restimulation with E7 peptide in the presence of Golgi inhibitor. (**C**) Memory phenotype of E7-specific CD8 T cells analyzed by flow cytometry in blood, spleen, and bone marrow at D45 post tumor implantation. (**D**) Complete responders were rechallenged at D75 with 10^5^ TC-1 cells and tumor growth was followed. Two-way ANOVA with Sidak’s multiple comparisons were used (* *p* < 0.05, ** *p* < 0.01, **** *p* < 0.0001) except in fig. A where Mann–Whitney test was used (* *p* < 0.05, ** *p* < 0.01). One representative of two independent experiments (*n* = 6–7).

The development and maintenance of a sustained antigen specific CD8 T cell response upon cancer vaccination is crucial to limit tumor immune escape. Thus, E7-specific CD8 T cell response in blood, spleen, and bone marrow were measured 45 days post TC-1 tumor implantation in KVK-vaccinated mice receiving NKG2A blockade therapy. Similar to the observations at earlier time point (Figure 3), NKG2A blockade significantly decreased the frequency of E7-specific CD8 T cells in all the three different compartments (Figure 4A) as well as the functionality of splenic E7-specific CD8 T cells (Figure 4B). In line with these results, the analysis of E7-specific CD8 T cells phenotype showed that combination with anti-NKG2A resulted in decreased frequency of effector CD8 T cells in blood, spleen, and bone marrow (Figure 4C). To investigate whether this effect was tumor-related, we analyzed peripheral immunogenicity and memory response in tumor-free mice. Similar to tumor-bearing mice, KVK vaccination in combination with anti-NKG2A resulted in reduced E7-specific CD8 T cells in the three peripheral compartments as well as the functionality of splenic antigen-specific CD8 T cells (Supplementary Figure S5A,B). Moreover, the analysis of memory response showed that anti-NKG2A decreased the amount of effector CD44^+^ KLRG1^+^ CD8 T cells and increased the proportion of central memory CD44^+^ CD62L^+^ CD8 T cells (Supplementary Figure S5C), suggesting that the effect of NKG2A is maintained in the long-term and it is not dependent from tumor presence.

Thus, we addressed whether this effect could have implications on long-term protection against homologous tumor rechallenge. Despite KVK vaccination in combination with anti-NKG2A resulted in complete eradication of primary tumor (Figure 1B), complete responders were only partially protected after tumor rechallenge. Indeed, while one out four mice rejected the reimplanted tumor (Figure 4D), the others displayed delayed tumor growth but were unable to control it in the longer term.

Taken together, these data show that anti-NKG2A modulates the systemic antigen-specific CD8 T cell response induced by KISIMA—VSV-GP-TAg vaccination decreasing its magnitude as well as the long-term immunological memory.

## 4. Discussion

The combination of protein-based cancer vaccine with oncolytic virus in heterologous prime-boost setting has been previously shown to elicit a robust and durable antigen-specific CD8 T cell response associated with a profound remodeling of the tumor microenvironment, resulting in strong tumor growth inhibition in mouse models [7,8].

Although intratumoral CD8 T cells are key players in controlling cancer progression, different counteracting mechanisms, including tumor antigen loss, reduced antigen presentation, and especially induction of T cell exhaustion, can limit their efficacy, leading to immune evasion [20]. In this study, we show that vaccine-induced tumor infiltrating antigen-specific CD8 T cells expresses different inhibitory receptors such as PD-1, Tim-3, and NKG2A already one week post boost. Despite the expression of these exhaustion markers, antigen-specific intratumoral CD8 T cells were able to secrete IFNγ, TNFα, and Granzyme B upon ex vivo restimulation, showing that they maintained their functionality, which was associated with tumor regression. Tumor analysis one month post boost vaccination, when tumors began to escape immune control, showed an increased portion of PD-1^+^Tim-3^+^NKG2A^+^ CD8 T cells and a reduction of cytokine secretion upon ex vivo restimulation compared to day 20, which goes along with the previously described multi-step modality of T cell exhaustion, progressively leading to attenuated CD8 T cell effector functions [21]. We previously reported that combination of heterologous prime-boost vaccination with a PD-1 blocking antibody partially restored T cell functionality resulting in prolonged efficacy [7]. Here, we focused on NKG2A, an inhibitory receptor mainly expressed on NK and CD8 T cells upon activation [22,23], whose interaction with its ligand, the non-classical MHC class I protein HLA-E (Qa-1b in mice), was shown to decrease cell cytotoxicity [14,15]. While most tissues express low basal levels, HLA-E is highly expressed by immune privileged sites including placenta and ductal epithelial cells of testis [24]. In cancers, HLA-E is frequently overexpressed, such as in non-small cell lung carcinoma [25], breast [26], renal carcinoma [27], and gynecological cancers [28], and high HLA-E expression is associated with worse prognosis.

NKG2A expression was shown to be significantly enhanced on intratumoral CD8 T cells by peptide-based cancer vaccination in mouse models [18]. We found that heterologous prime-boost using a protein-based vaccine and an oncolytic virus increased NKG2A expression on both intratumoral and circulating antigen-specific CD8 T cells, but not on NK cells. Interestingly, the expression kinetic differed between the two compartments, as NKG2A was already upregulated by tumor-infiltrating antigen-specific CD8 T cells 24 h post VSV-GP-TAg boost, whereas in the blood overexpression was observed only one week later. This difference might be related to the abundance of antigen presentation within the tumor, as repeated target engagement was recently reported to be required for NKG2A expression on T cells [29]. Increased NKG2A expression in blood could derive from recirculation of intratumoral CD8 T cells or could be induced by replication of VSV-GP-TAg in the spleen, which could result in higher antigen concentration and presentation, and NKG2A upregulation by peripheral CD8 T cells. Supporting this last hypothesis, upon homologous protein-based vaccination we observed NKG2A overexpression on intratumoral but not circulating antigen-specific CD8 T cells.

Targeting NKG2A with a blocking antibody has been shown to restore NKG2A^+^ CD8 T cell functionality and cytotoxic capacity in different mouse models [16,19], and its human application is currently assessed in several clinical trial in combination with other immunotherapies [19,30]. We found that anti-NKG2A significantly reduces the exhaustion of intratumoral CD8 T cells induced by heterologous prime-boost vaccination resulting in lower frequency of PD-1^+^Tim-3^+^NKG2A^+^ cells, which was associated with prolonged tumor control and increased mouse survival. These data are consistent with recent studies showing anti-NKG2A as a combinatory treatment to potentiate the antitumoral efficacy of peptide-based cancer vaccines in mouse models [18,19]. Surprisingly, the reduced intratumoral CD8 T cell exhaustion upon anti-NKG2A treatment did not increase CD8 T cell activation nor ex vivo functionality, both in the tumor remission phase and later during tumor relapse. Effect of anti-NKG2A has been reported to rely on both NK and CD8 T cells, depending on tumor models [18,19]. While KISIMA—VSV-GP-TAg vaccination induced a strong tumor infiltration of NKG2A^+^ antigen-specific CD8 T cells, only limited tumor infiltration of NK cells and no up-regulation of NKG2A expression was observed, suggesting that combination effect could rely mainly on CD8 T cells.

Immune check point blockade (ICB) has revolutionized the field of immunotherapy and is proving highly effective in a still narrow patient population [31]. However, even patients responding to treatment often develop resistance and relapse [32]. Different mechanisms have been associated with resistance to ICB therapy, both tumor intrinsic, such as antigen loss or defect in antigen processing and presentation, and tumor extrinsic, such as modulations of the tumor microenvironment (TME) increasing immune suppression [33,34,35]. We found that NKG2A blockade significantly modified KV-induced systemic T cells response, reducing the frequency and functionality of circulating antigen-specific CD8 T cells. The impairment of CD8 T cell response did not depend on the source of antigen, as it was observed with antigens of viral (HPV-E7 and VSV-GP) origin as well as tumor specific neoantigens (adpgk, reps-1, rpl-18), which exhibit a wide range of immunogenicity. Moreover, NKG2A blockade reduced the development of an effective peripheral memory response essential to confer long-term immunological protection.

Phenotypically, NKG2A combination shifted vaccination-induced memory CD8 T cells towards central memory rather than effector. Notably, this effect was observed also in tumor-free mice demonstrating the tumor-independent capacity of anti-NKG2A to shape memory immune response.

The effects observed on systemic response might be related to treatment schedule; here we determined that anti-NKG2A treatment should start according to its target upregulation on vaccine induced antigen-specific CD8 T cells; however, it was previously reported that different scheduling of anti-PD-1 could have the opposite impact on the efficacy of cancer vaccine in preclinical models [2], while in clinical studies ICB is combined with other immunotherapy using a variety of scheduling. Interestingly, the effect on immunogenicity was observed during anti-NKG2A treatment and was maintained after treatment termination, suggesting that a short-term treatment was sufficient to amend the immune response elicited by vaccination. Further studies would be required to assess the impact of different anti-NKG2A treatment regimens on the development of CD8 T cell responses upon vaccination.

## 5. Conclusions

Our study reveals the dual effect of anti-NKG2A on vaccine-induced antitumoral CD8 T cell immune response in a compartment-dependent manner. While NKG2A blockade potentiates intratumoral CD8 T cell efficacy, resulting in improved tumor growth control and mouse survival, it limited the differentiation of effector memory CD8 T cells in periphery. Overall, these data show that NKG2A blockade differently influences systemic and intratumoral vaccine-induced CD8 T cell immune response, suggesting that the analysis of multiple compartments is helpful in order to elucidate the outcome of combinatory immunotherapy.

## Data Availability

The data presented in this study are available on request from the corresponding author.

## Supplementary Materials

The following supporting information can be downloaded at: https://www.mdpi.com/article/10.3390/cancers16112036/s1, Table S1: KISIMA and VSV-GP constructs with epitope sequences used in the respective tumor models. Figure S1: Heterologous prime-boost KV vaccination enhances NKG2A expression on peripheral and intratumoral antigen-specific CD8 T cells. Figure S2: Combination treatment with anti-NKG2A reduces KV-induced exhaustion of intratumoral antigen-specific CD8 T cells at later time point. Figure S3: Effect of the combination treatment of KISIMA-VSV-GP-TAg vaccination with aNKG2A in MC-38 tumor model. Figure S4: NKG2A blockade in combination with KV reduces antigen-specific CD8 T cells in the periphery of MC-38 tumor bearing mice. Figure S5: anti-NKG2A decreases KVK-induced long-term antigen-specific CD8 T cell response in periphery of tumor-free mice. Figure S6: gating strategy used for the analysis of CD8 T cell exhaustion by flow cytometry. Figure S7: Gating strategy used for the analysis of the cytokine production after ex vivo restimulation with E7-peptide. Figure S8: Gating strategy used for the analysis of memory phenotype by flow cytometry.
