# Supplementary material for: Bimodal Effect of NKG2A Blockade on Intratumoral and Systemic CD8 T Cell Response Induced by Cancer Vaccine
Source: Cancers (Basel). 2024 May 27;16(11):2036. doi: 10.3390/cancers16112036 (PMC11171001; doi:10.3390/cancers16112036)
Supplement: Supplementary file 1 [file cancers-16-02036-s001.zip › cancers-3018562-supplementary.pdf]

KISIMA	VSV	Epitopes	Sequence	Tumor model
KISIMA-Mad25	VSV-GP-HPV	HPV-E7 CD8	RAHYNIVTF	TC-1
KISIMA-Mad46	VSV-GP-Mad46	Adpgk<br>Reps1<br>Rpl18	ASMTNMELM<br>AQLANDVVL<br>KILTFDRL	MC-38

Table S1. KISIMA and VSV-GP constructs with epitope sequences used in the respective tumor models.

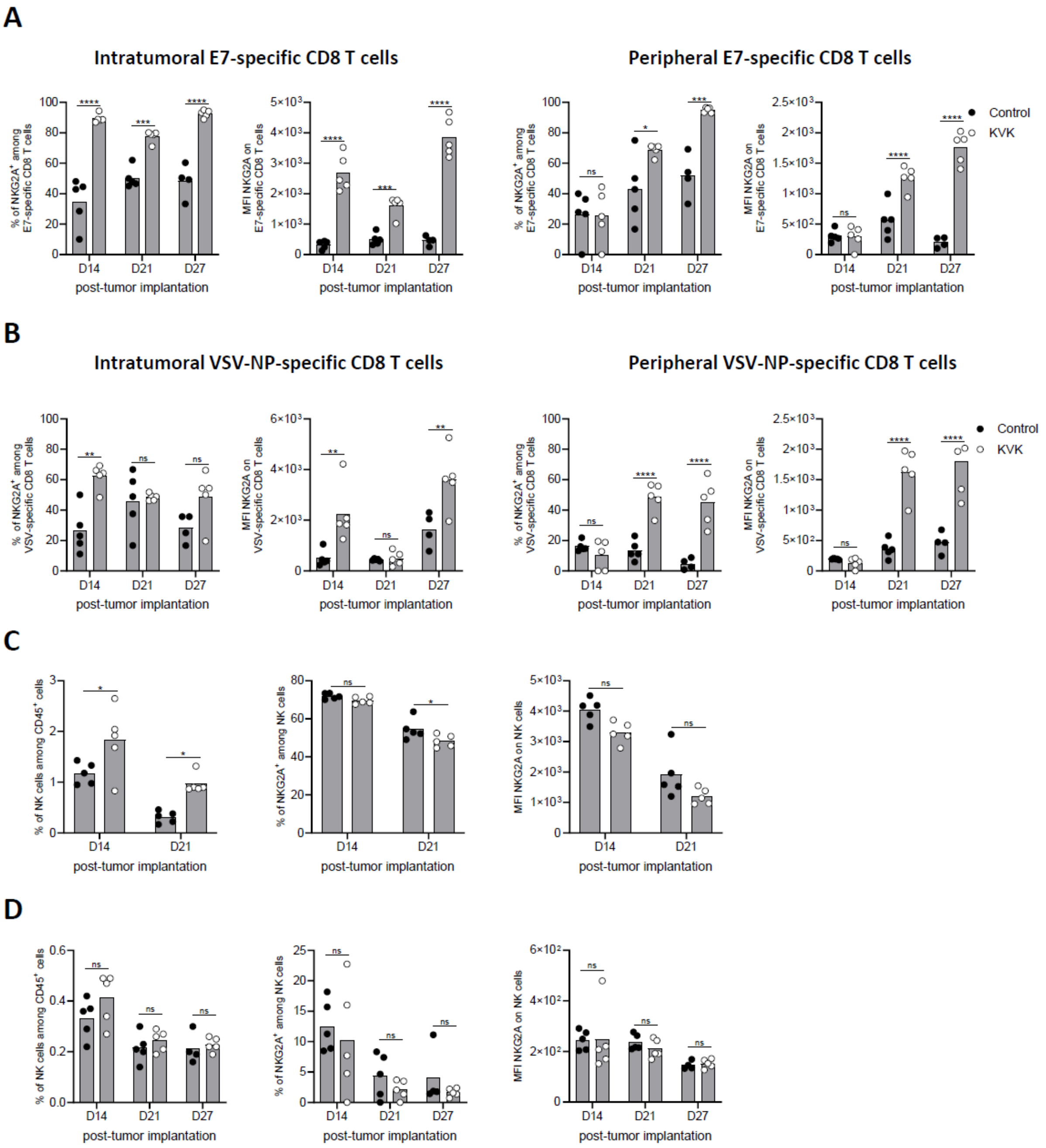

Figure S1. Heterologous prime-boost KV vaccination enhances NKG2A expression on peripheral and intratumoral antigen-specific CD8 T cells. 10^5 TC-1 cells were implanted s.c. on the back of C57BL/6J mice at D0. Six days later, mice were primed with KISIMA-Mad25 vaccine followed by a second boost with VSV-GP-HPV. The expression of NKG2A was measured by flow cytometry on antigen specific CD8 T cells isolated from blood and tumors at different time post tumor implantation. (**A**) Frequency and MFI of NKG2A$^+$ cells among E7-specific CD8 T cells in the tumor and blood after heterologous prime-boost vaccination. (**B**) Frequency and MFI of NKG2A$^+$ cells among VSV-specific CD8 T cells in the tumor and blood after heterologous prime-boost vaccination. (**C**-**D**) Frequency of NK cells, frequency and MFI of NKG2A$^+$ cells among NK cells in the tumor (**C**) and blood (**D**). Two-way ANOVA test with Sidak's multiple comparisons was used. *p<0.05, **p<0.01, ***p<0.001, ****p<0.0001. MFI: mean fluorescence intensity. One representative of two independent experiments (n=5 for each time point).

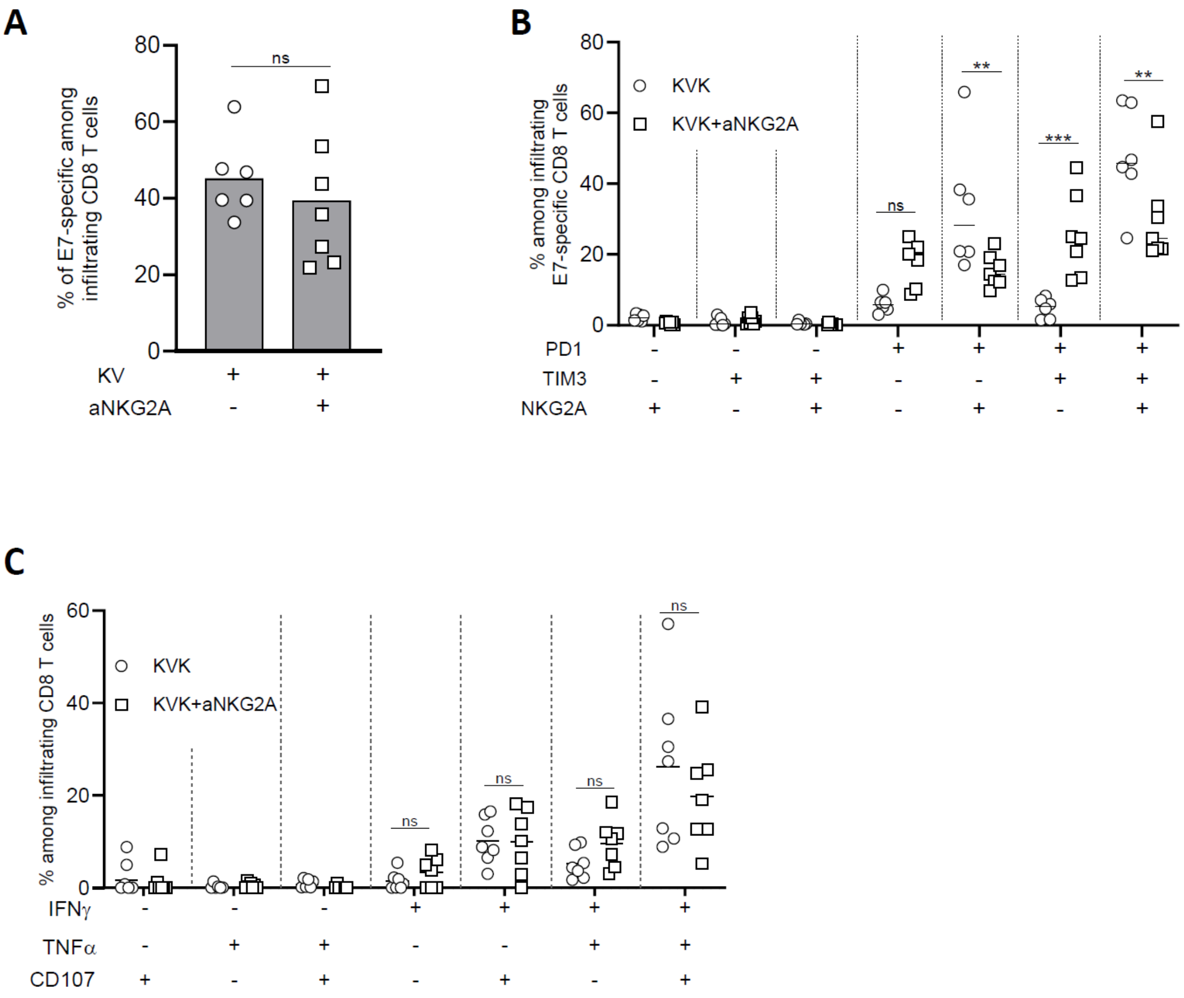

Figure S2. Combination treatment with anti-NKG2A reduces KV-induced exhaustion of intratumoral antigen-specific CD8 T cells at later time point. TC-1 tumor-bearing mice were vaccinated with KISIMA-Mad25 vaccine at D6 post tumor implantation followed by a boost with VSV-GP-HPV at D13 and a second boost with KISIMA at D27. Mice were treated with anti-NKG2A starting 4 days post VSV-GP-HPV injection for a total of five injections. Mice were sacrificed at D45, tumors were harvested, processed and CD8 T cell immune response was analyzed by flow cytometry. (**A**) Frequency of E7-specific among CD8 T cells in the tumor at D45 post tumor implantation (**B**) Frequency of E7-specific CD8 T cell expressing exhaustion markers (**C**) Cytokine production by antigen-specific CD8 T cells in the tumor was measured by intracellular staining after *ex-vivo* restimulation with E7-peptide. Two-way ANOVA test with Sidak's multiple comparisons was used **p<0.01, ***p<0.001.

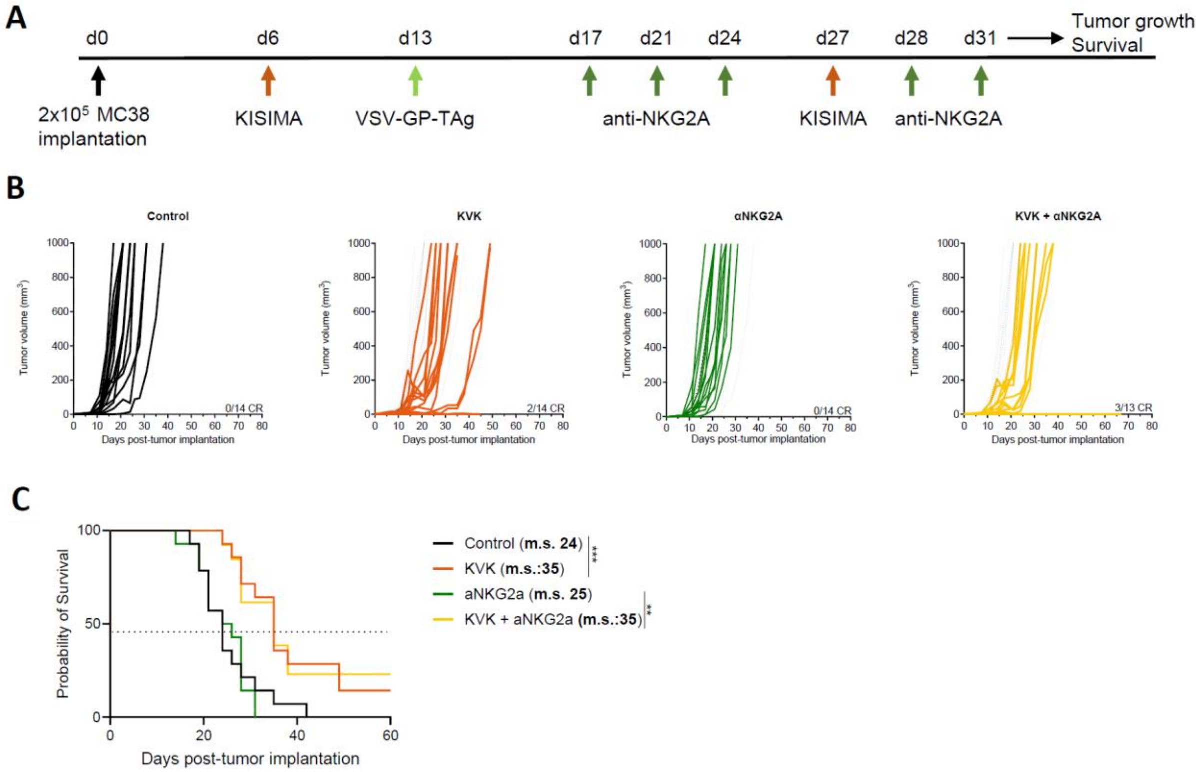

Figure S3. Effect of the combination treatment of KISIMA-VSV-GP-TAg vaccination with aNKG2A in MC-38 tumor model (**A**) Experimental schedule. MC-38 tumor-bearing mice were vaccinated with KISIMA-Mad46vaccine at D6 post tumor implantation followed by a boost with VSV-GP-Mad46 at D13. A further boost with KISIMA-Mad46 was administered at D27. Mice were treated with aNKG2A starting 4 days post VSV-GP-Mad46 boost for a total of 5 injections. Tumor growth (**B**) and survival (**C**) were monitored. CR, complete regression. ms, median survival. Long-rank Mantel-Cox test. **p<0.01, ***p<0.001. Data are pooled from two independent experiments (Control n=14, KV n=14, aNKG2A n=14, KV+aNKG2A n=13).

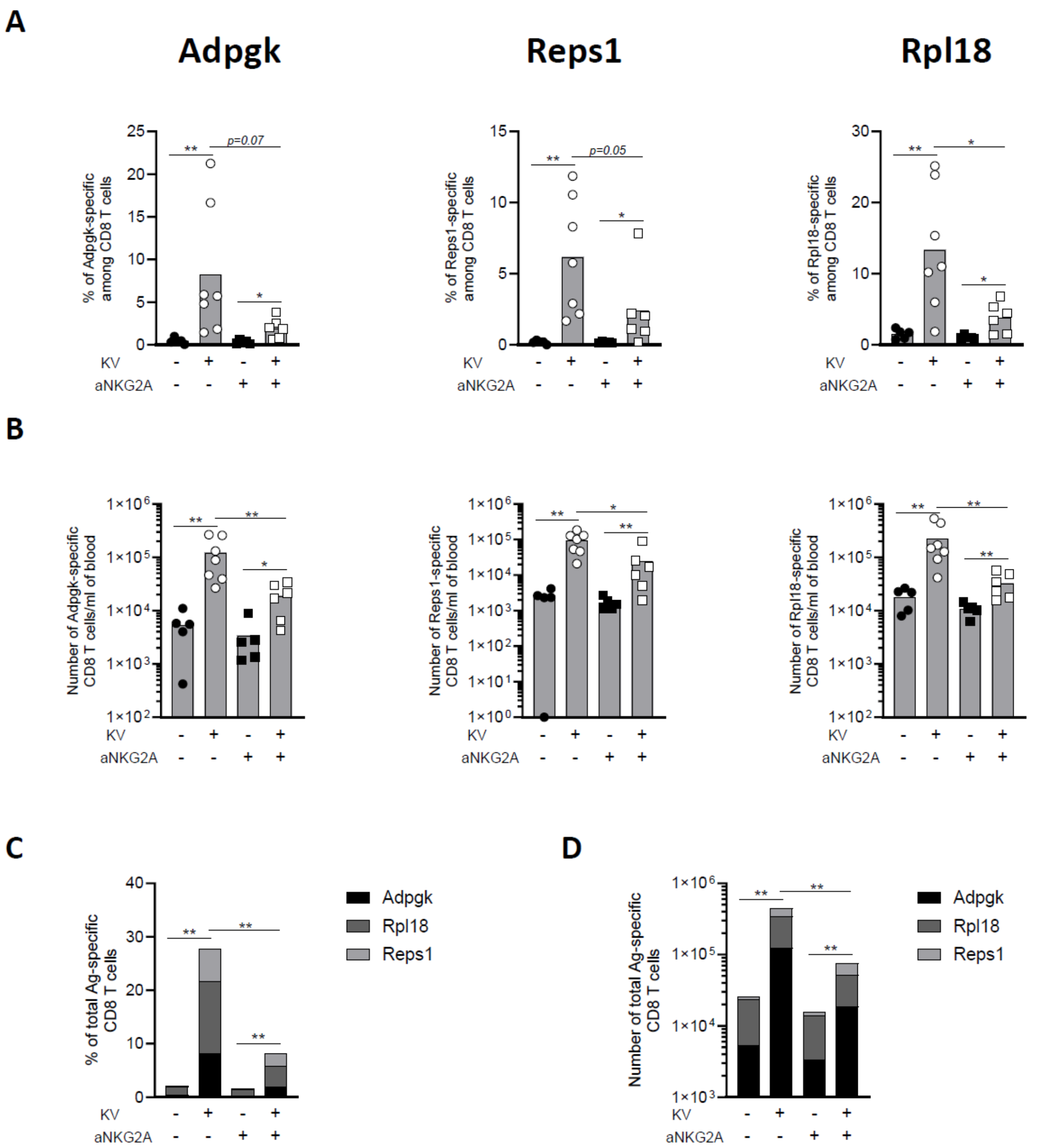

Figure S4. NKG2A blockade in combination with KV reduces antigen-specific CD8 T cells in the periphery of MC-38 tumor bearing mice. MC-38 tumor-bearing mice were vaccinated with KISIMA-Mad46 vaccine at D6 post tumor implantation followed by a boost with VSV-GP-Mad46 at D13 and a second boost with KISIMA-Mad46 at D27. Mice were treated with anti-NKG2A starting 4 days post VSV-GP-Mad46 boost for a total of 5 injections. One week post VSV-GP boost, blood was collected, and CD8 T cell response was analysis by flow cytometry. (**A-B**) Frequency (**A**) and number (**B**) of Adpgk, Reps1 and Rpl18- specific CD8 T cells measured in the blood one week post VSV-GP-TAg boost. Mann-Whitney test was used *p<0.05,**p<0.01. (**C-D**) Frequency (**C**) and number (**D**) of total antigen-specific CD8 T cells measured in the blood one week post VSV-GP-TAg boost. Two way ANOVA was used ****p<0.0001. One representative of two independent experiments (Control n=5, KV n=7, aNKG2A n=5, KV+aNKG2A n=6).

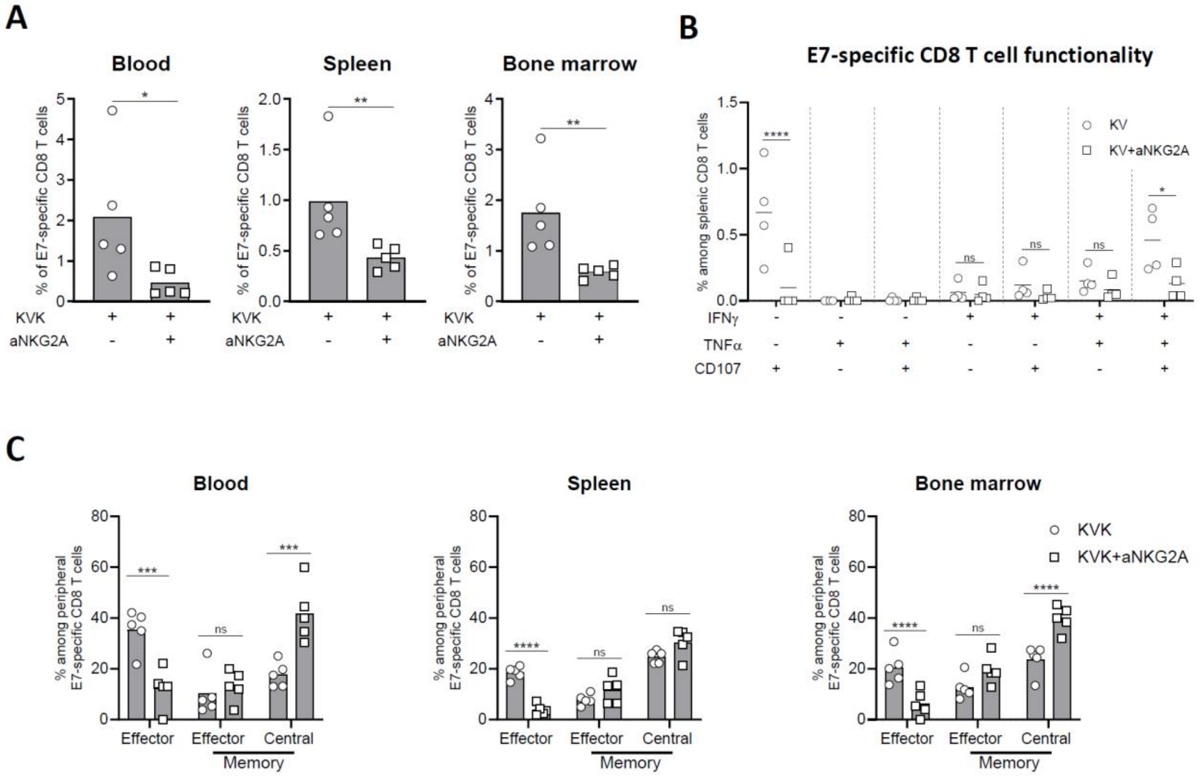

Figure S5. anti-NKG2A decreases KVK-induced long-term antigen-specific CD8 T cell response in periphery of tumor-free mice. C57BL/6J mice were vaccinated with KISIMA-Mad25 vaccine at D0 followed by a boost with VSV-GP-HPV at D13 and a second boost with KISIMA at D27. Mice were treated with anti-NKG2A starting 4 days post VSV-GP-HPV injection for a total of five injections. Mice were sacrificed at D91, tumor, spleen and bone marrow were harvested, processed and CD8 T cell immune response was analyzed by flow cytometry. **(A)** Frequency of E7-specific among CD8 T cells in blood, spleen and bone marrow of tumor free mice at D91 post VSV-GP-HPV boost (**B**) Cytokine production by antigen-specific CD8 T cells in the spleen was measured by intracellular staining after *ex-vivo* restimulation with E7 peptide. (**C**) Memory phenotype of E7-specific CD8 T cells analyzed by flow cytometry in blood, spleen and bone marrow at D91 post VSV-GP-HPV boost. Mann-Whitney test was used (*p<0.05,**p<0.01,***p<0.001, ****p<0.0001) except in fig B where Two-way ANOVA with Sidak's multiple comparisons were used (*p<0.05,**p<0.01,***p<0.001, ****p<0.0001). One representative of two independent experiments (n=5).

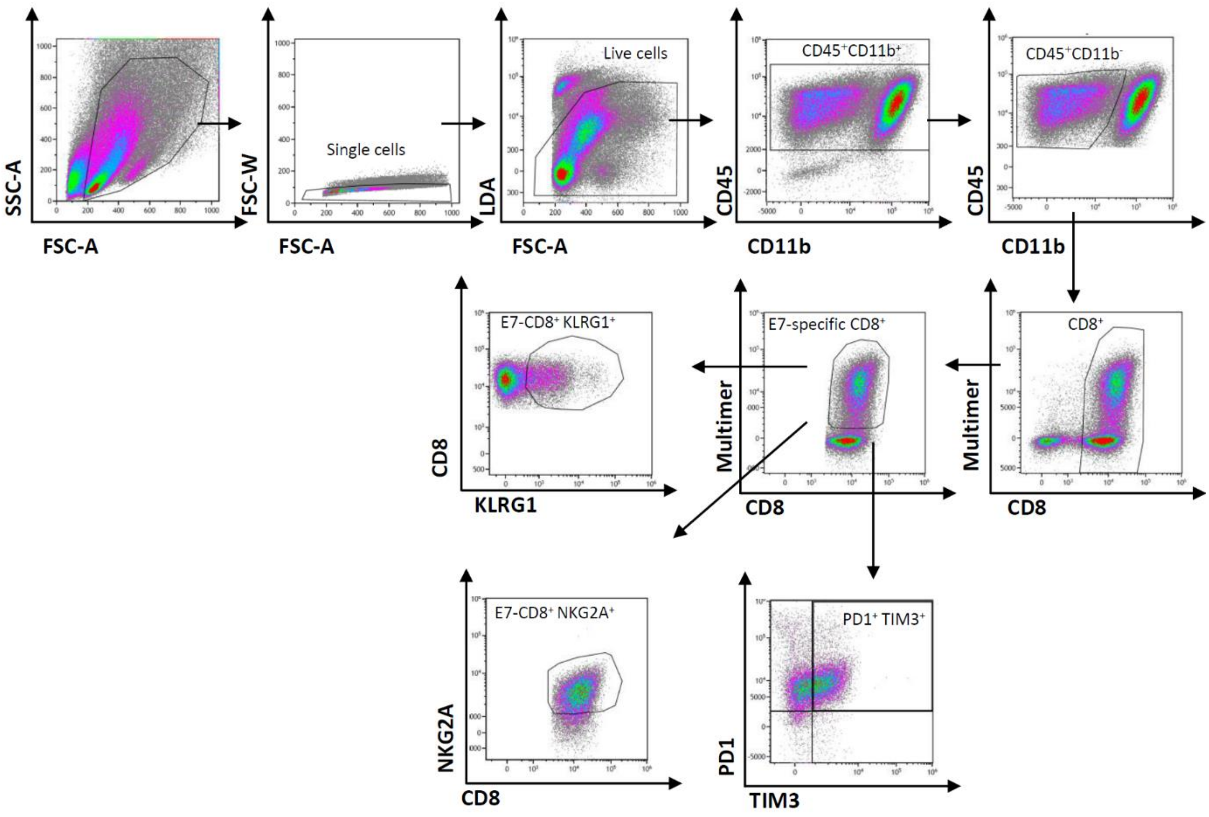

Figure S6. Gating strategy used for the analysis of CD8 T cell exhaustion by flow cytometry.

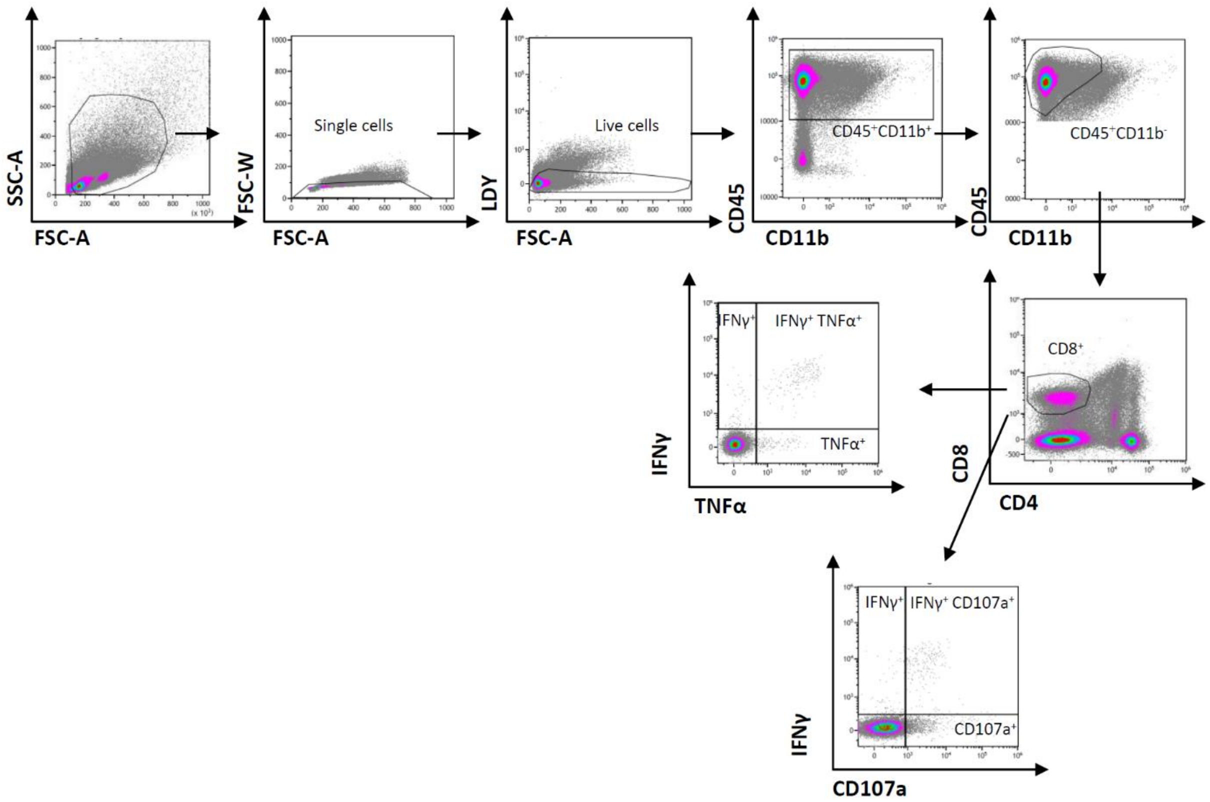

Figure S7. Gating strategy used for the analysis of the cytokine production after *ex vivo* restimulation with E7-peptide.

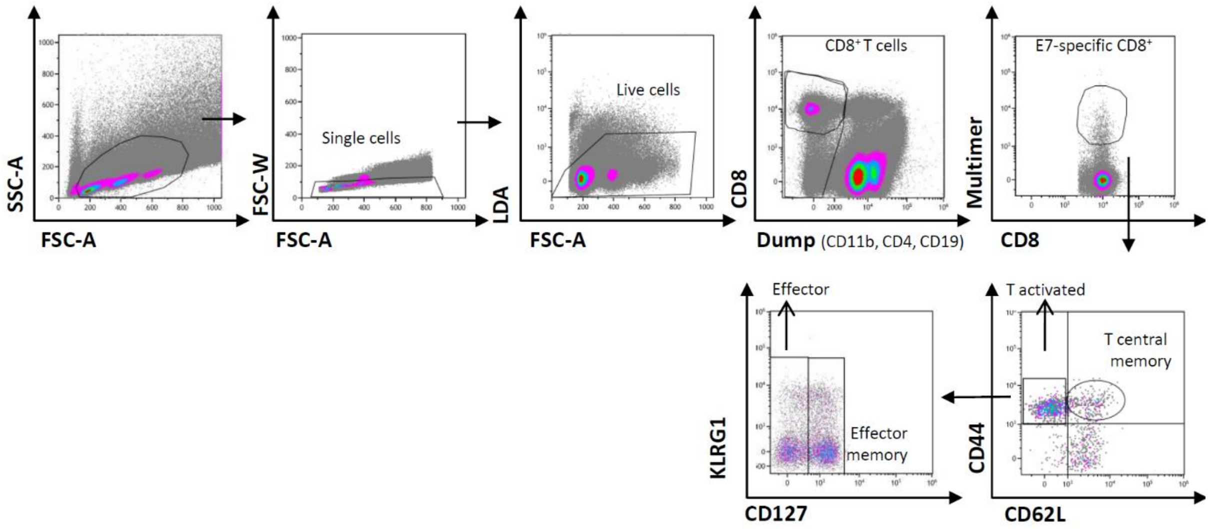

Figure S8. Gating strategy used for the analysis of memory phenotype by flow cytometry.
